# Implementation of integrated knowledge translation in NCD research: Examining intervention components

**DOI:** 10.1093/eurpub/ckac131.546

**Published:** 2022-10-25

**Authors:** K Sell, EA Rehfuess, LM Pfadenhauer

**Affiliations:** 1 IBE, LMU Munich, München, Germany; 2 PSPH, München, Germany

## Abstract

**Background:**

Integrated knowledge translation (IKT) has been a cornerstone of the Collaboration for Evidence-based Healthcare and Public Health in Africa (CEBHA+) with partners in Ethiopia, Germany, Malawi, Rwanda, South Africa, and Uganda. The consortium conducts research on preventing and treating non-communicable diseases as well as road traffic injuries. IKT is understood as the continuous engagement of decision-makers throughout the research process in order to build equitable, mutually beneficial partnerships to conduct policy-relevant research and, ultimately, strengthen evidence-informed decision-making (EIDM). Gradually, a structured “CEBHA+ IKT approach” was developed, including systematic stakeholder mapping and analysis, and the development of local IKT strategies.

**Methods:**

We conducted a mixed-methods process and outcome evaluation of this IKT intervention. This comprised structured interviews, an online survey, and document analyses at two time points, two and four years after IKT initiation.

**Results:**

Preliminary results show that partnerships with decision-makers were successfully established or strengthened. While continuous engagement was implemented, fidelity to formalised IKT strategies was variable. The development, monitoring and updating of the IKT strategies, originally conceptualised as an essential intervention component, has been helpful for some CEBHA+ researchers and may facilitate implementation. However, the vision for decision-maker engagement as well as emphasis on continuous engagement (defined as a deliverable) emerged as more important intervention drivers and may be conceptualised as intervention components.

**Conclusions:**

A strong vision and continuous engagement with decision-makers are critical for strengthening EIDM. Formalised IKT strategies proved to be of moderate importance in current CEBHA+ research activities, but may turn out to be an essential intervention component if implemented from the start of a research project.

**Key messages:**

• Continuous engagement with decision-makers has successfully strengthened or established partnerships between researchers and decision-makers.

• Formal IKT strategies to plan stakeholder engagement were developed but continuous stakeholder engagement and a strong vision proved to be of greater importance.

